# Linear Motor Driven Leg-Press Dynamometer for Testing, Training, and Rehabilitation: A Scoping Review with a Focus on the Concept of Serial Stretch Loading

**DOI:** 10.3390/ijerph19084445

**Published:** 2022-04-07

**Authors:** Ján Cvečka, Matúš Krčmár, Dušan Hamar, Helmut Kern, Christian Hofer, Stefan Löfler, Matej Vajda

**Affiliations:** 1 Faculty of Physical Education and Sports, Hamar Institute for Human Performance, Comenius University in Bratislava, 81469 Bratislava, Slovakia; jan.cvecka@uniba.sk (J.C.); matej.vajda@uniba.sk (M.V.); 2 Faculty of Education, Department of Physical Education and Sports, Constantine the Philosopher University in Nitra, 94901 Nitra, Slovakia; 3 Faculty of Physical Education and Sports, Department of Biological and Medical Sciences, Comenius University in Bratislava, 81469 Bratislava, Slovakia; dusan.hamar@uniba.sk; 4 Ludwig Boltzmann Institute for Rehabilitation Research, 1140 Vienna, Austria; helmut@kern-reha.at (H.K.); christian.hofer@ottobock.com (C.H.); stefan.loefler@rehabilitation.lbg.ac.at (S.L.)

**Keywords:** proprioception, isokinetic, strength, power, musculoskeletal injuries

## Abstract

Background: The purpose of this scoping review was to analyze the evidence of acute and long-term effects of the application of leg-press strength training with or without serial stretch-loading stimuli on various biomechanical and physiological outcomes. Methods: This review was performed in accordance with PRISMA for Scoping Reviews recommendations, and two researchers independently searched the following databases: PubMed, Web of Science, Scopus, ScienceDirect, ProQuest, Cochrane, and Google Scholar. All studies that used unique leg-press device for testing, acute responses and long-term adaptation were included in this review, irrespective of the measured outcomes. A total of 13 studies were included in this review, with 5 focused on the testing capabilities of the device and acute training responses and 8 focused on the long-term adaptations in various physical and physiological outcomes. Results: Regarding the acute responses after leg-press strength training with or without serial stretch-loading stimuli, visible changes were observed in the muscle force, rate of force development, and hormonal concentrations between pre- and postmenopausal women (only one study). Long-term studies revealed different training adaptations after performing leg-press strength training with unique serial stretch-loading stimuli. A positive trend for leg-press strength training with serial stretch-loading was recorded in the young population and athletes; however, more variable training effects favoring one or the other approach were achieved in the older population. Conclusions: In summary, this review shows the uniqueness and usability of a leg-press device that is capable of various exercising modes, including special serial stretch-loading stimuli. The use of this device can serve as a positive addition to training regiments, and the main application appears to be suitable for rehabilitation needs.

## 1. Introduction

**Figure 1:**
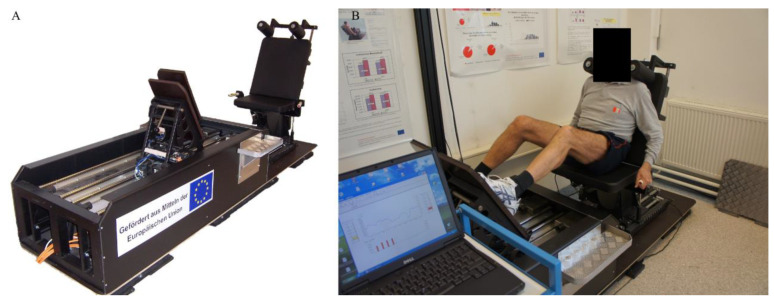
Represents unique linear motor-driven leg press dynamometer (**A**) and position during testing/training (**B**).

Currently, using the terms “machine” or “training device” in reference to training and rehabilitation is somewhat controversial and/or sensitive for many practitioners from many areas of sports training and medicine. Some object to the nonfunctionality of these devices, while others use these devices during training alone or during the rehabilitation process. However, in both the abovementioned areas of sports training and medicine, the employment of machines is widely accepted and can play an important role in various situations. For instance, before and after operation, injured athletes noticed various deficits in addition to the safer and more controllable environments during complex solution processes [1]. When referencing the term ‘machine’, we must understand that these machines have progressed over time and are now very sophisticated, with multiple functions, modes, and outcomes, especially in terms of rehabilitation, where they accelerate recovery after injuries, operations, and other health-related complications [2]. In particular, robots are frequently applied for the rehabilitation of upper and lower extremities, and they can include grounded and wearable exoskeletons and grounded end-effector devices for controlling single or multiple joints. However, this area requires further exploration due to the limited number of studies [3]. Among many other sophisticated machines, our laboratory has developed in collaboration with the University of Vienna a linear motor-driven leg press dynamometer (Figure 1) that presents a unique serial stretch loading mode that allows for the generation of force peaks during exercise. The next part of this review is focused directly on this unique device.

Strength and power are two factors that affect sports performance, and they are also the subject of wider research by many researchers, mainly in connection with the elderly population and/or rehabilitation [4,5,6], which is one of the reasons that led us to build a unique linear motor-driven leg press dynamometer. The main aim was to build a diagnostic and training device that could be used for multiple purposes in both younger and older subjects as well as for rehabilitation. The uniqueness of this device lies in the fact that it can generate force peaks by rapid changes in the direction or velocity of the movement during the concentric and eccentric phases of the movement.

To construct our prototype, we used two Kollmorgen IC 2022 linear motors (Danaher Corp., Washington, DC, USA). The linear engine represents a classic electric motor whose stator is deployed in the form of a so-called “magnetic path”, with a length of 120 cm (maximum range of motion 80 cm plus engine length 40 cm). The engine is capable of producing a maximum force of 1800 N in both the active and passive breaking modes. If the duration of the load exceeds 4 s, then it can generate active force or passive resistance up to 800 N. The motor can accelerate at 15 g, which is equivalent to 147 m/s^2^. Both motors are powered by special power supplies (SERVOSTAR, Kollmorgen, IL, USA). For operational purposes, it was necessary to capture the actual force and position at a frequency of 1000 Hz. The velocity of the movement is calculated by deriving the change in position over time, and the power output is calculated as the product of the velocity and force. Force capture is carried out using dynamometers on the strain gauge mounted to stainless steel, and they have a range of up to 4000 N and a resolution of less than 1 N. The dynamometer was positioned to connect the pedal (where the foot is placed) and the linear engine. A linear incremental encoder (RGH 22, Renishaw, Bedford, UK) with a resolution of 0.001 mm was used to sense the position. A high frequency of data collection (e.g., position, velocity, or force development) and computer control of the linear engines makes this system very unique and universal, which means that it is able to work in different modes, including constant resistance, constant velocity, and isometric mode. In the next part of this review, the abovementioned modes with the unique serial stretch loading concept will be explained.

Except for the unique serial stretch mode, which will be explained later in this section, this dynamometer offers all known modes, including modes with constant resistance (isoinertial mode), constant velocity (isokinetic mode), and isometric contraction (isometric mode). Constant resistance is based on constant engine resistance, regardless of the force exerted by an individual. Therefore, if the force exerted does not reach the level of preset resistance, the pedal remains in its baseline position. For instance, if the preset resistance corresponds to 1000 N, pedals stay in their baseline position and start to move only after the force exerted by the individual exceeds this value. Isometric mode can be used independently with constant resistance mode or constant velocity (isokinetic) mode. The isometric mode can be adjusted by using the constant resistance mode with the resistance set well over the value a person can produce at maximal effort. Due to the high sampling frequency (1000 Hz), the system enables us to measure not only the maximal force production but also the rate of force development. The isokinetic mode uses tight feedback regulation of breaking (concentric phase) or driving (eccentric phase) forces and maintains the movement velocity at a preset level. Additionally, the duration of the acceleration phase at the beginning and deceleration phase at the end of the concentric and eccentric phases as well as the starting position can be set.

The setting of an isokinetic mode can create serial stretch loadings characterized by repeated force peaks over the level that can be achieved by similar effort during isokinetic movement. Peak forces during the concentric phase are elicited by short (5 mm) backward movements. The rationale for this behavior is based on the physiological principle that force generated by a muscle decreases with increasing velocity and vice versa. In other words, to increase force production, the velocity needs to be decreased, stopped, or reversed [4,7]. However, increasing velocity is not applicable in the eccentric phase, because according to the Hills curve, this would lead to a decrease in force production [4]. To elicit force peaks in the eccentric phase, one needs to impose short accelerated eccentric movement.

The system also allows us to set other variables that affect force peaks, such as the rapidness of velocity changes and the frequency of such cycles [4]. Actually, this functionality represents the originality of the device and can be considered a specific or unique mode that may increase the efficiency of performance-oriented strength training in elite athletes but also ameliorate strength deficits in patients after injuries or in pre- and postoperative conditions. In addition, in all of the modes mentioned previously, the pedals of the device can move independently of each other, which means that an individual can be trained or tested in unilateral mode (the same parameters can be obtained as during bilateral training or testing). Given the mentioned modes, this device offers extensive adjustment of the range of motion during training and testing from 0° (full extension) up to ±140° (full flexion). However, these functions also depend on the individual’s capacity.

According to all of the abovementioned advantages of the device, it can serve as a general rehabilitation and diagnostic tool that may improve several muscular functions, including maximal muscle force (concentric/eccentric/isometric), muscular power output, and rate of force development. Some results from our laboratory indicate that using the unique stretch loading during leg press exercise may have a more favorable effect compared to standard loading. However, these results need to be verified by an in-depth analysis of multiple studies. In the next part of this review, we will focus on acute and long-term adaptation using this unique device. It should be mentioned that this device was patented (Patent Nr.: AT 505 722 A1 15 March 2009), and its use is limited due to the low number of manufactured pieces. Regardless, we performed a literature search to find all relevant studies and possibly similar devices to perform a comprehensive analysis of all available resources to assess the impact of the linear motor-driven leg press dynamometer using a unique concept of serial stretch loading from acute testing to short- or long-term training perspective on various health-related and performance outcomes.

## 2. Materials and Methods

### 2.1. Literature Search

**Figure 2:**
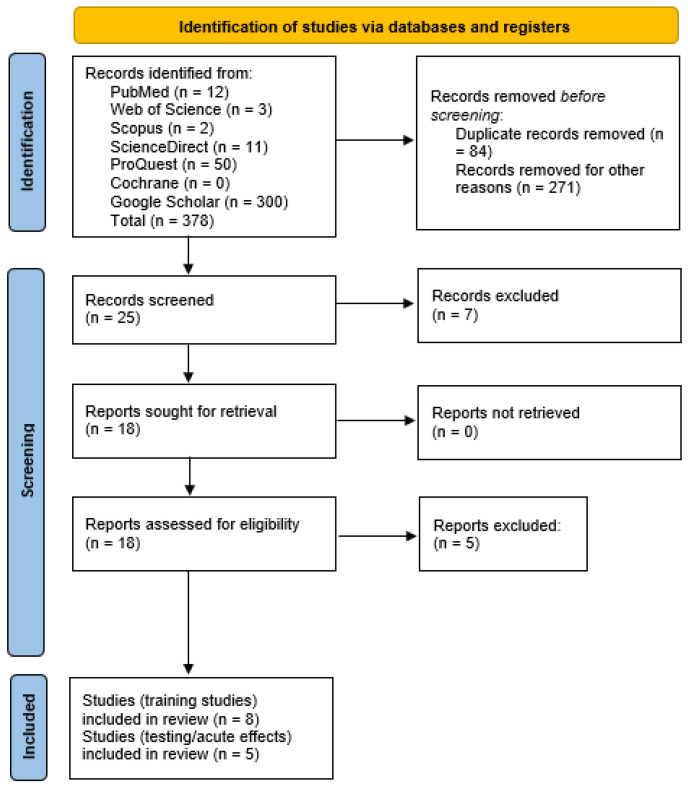
Flow diagram representing the selection process.

A database search was conducted in April and May 2021 based on PRISMA (Preferred Reporting Items for Systematic reviews and Meta-Analyses extension for Scoping Reviews—PRISMA-ScR) guidelines [8]. All studies were checked by two reviewers (M.K. and M.V.). Studies were searched in the following databases: PubMed, Web of Science, Scopus, ScienceDirect, ProQuest, Cochrane, and Google Scholar. The combination of keywords during the search process consisted of (“serial stretch loading” OR “serial stretch stimuli”) AND (strength OR power OR performance OR exercise OR resistance OR force OR rehabilitation OR muscle mass OR muscle damage) AND (leg press OR leg extension OR exercise device). Only original articles published in English or German (due to the originality of the review and proposed device) were retained. After the initial check, duplicate studies were removed based on the selection criteria, and the eligibility of the remaining studies was screened. Finally, the remaining studies were fully read and included in this review. In addition, the reference section of each article was also examined for potential missing articles that were not found in the database search. The chart representing the selection process is shown in Figure 2.

### 2.2. Inclusion/Exclusion Criteria

The criteria used to identify articles for inclusion are as follows: healthy adults (>18 years old); inclusion of leg press exercises or similar exercises in conjunction with the concept of serial stretch loading applied during acute and testing periods or over long-term periods; publication in peer reviewed journal in English and potentially in German (inclusion of domestic sources is also allowed due to the complexity and originality of the mentioned device, where the detailed construction descriptions are in the domestic language); inclusion of neuromuscular performance-related and physiological-related parameters; and submission to a review procedure within the journal where they are published (this must be stated in the journal instructions or supported by available review documents).

The exclusion criteria were as follows: training studies that did not use the concept of serial stretch loading during machine-based exercise; and a lack of neuromuscular performance-related and physiological-related parameters.

During the database search, special attention was focused on linear motor-driven leg press devices with the possibility of producing serial stretch loading. There was no limit to the search domain regarding the participant training level, exercise modalities and characteristics, intervention designs, or measured physiological and neuromuscular outcomes.

Due to the low number of selected studies, which is mainly limited by the originality of the leg press device, laboratories that use this device, only scoping reviews without meta-analysis are presented.

### 2.3. Quality Assessment—PEDro Scale

The eleven-point PEDro scale (preferred reporting items for systematic reviews and meta-analysis) [9] was used to evaluate the methodological quality of the studies. Two authors (M.K. and M.V.) carried out the qualitative assessment, with any disagreements resolved by mutual consensus. The first point regarding external validity was not used to calculate the PEDro scale. From the remaining items, we excluded 5, 6, and 7 because the participants and assessors could not be blinded in such studies. The highest possible score was 7, and the rating scale was as follows: 0–3 = “poor quality”, 4 = “moderate quality”, 5 = “good quality”, and 6–7 = “excellent quality” based on previous intervention reviews [10]. The quality results of the individual studies are shown in Table 1. Due to the focus of this review and the selection of individual studies (training studies as well as acute/testing studies), high-quality evaluations were not included in all of them. However, these studies were included in this review due to the versatility of the leg press dynamometer, and they are presented separately in the Section 3 and Table 2.

## 3. Results

Table 1 shows the qualitative assessment of each study based on the adjusted PEDro scale. Regarding acute/testing studies (right part of Table 1), only 5 studies were included in this review. Qualitative assessments of these studies were somewhat variable, with 1 study receiving 2 points (poor quality) and 2 studies receiving 6 points each (excellent quality). The lowest score was achieved for the study by Billy et al. [12], which was primarily because of the cross-sectional study design of this study. It should be noted that studies with lower scores were nevertheless retained in this review because the study focus was on the wide possibilities of the presented motor-driven leg press device. Regarding the training studies (left part of the table), only 1 study presented a poor score due to the absence of some key elements during evaluation. Table 2 and Table 3 show the acute/testing and training studies, respectively. Only studies where leg press devices were used, either for acute effects and testing or training, were included. The tables indicate the very wide possibilities and usability of the leg press device. Special attention was given to the SSL mode (serial stretch loading) for the training and acute studies. The results of selected studies are not limited to selected outcomes, and all of the most important findings from each study are presented because of the high variability of individual studies and outcomes that were examined (it would not be possible to consolidate several studies with the same outcome). Moreover, the main goal of this review was not to focus on selected outcomes but rather to provide the wide range of possibilities of this device.

## 4. Discussion

The purpose of this scoping review was to introduce a unique motor-driven leg press dynamometer and its versatility and usability for testing and training and potentially for future rehabilitation purposes. The advantage of this device lies in the number of modes that can be adjusted separately as well as combined. However, the main uniqueness of this device lies in the SSL mode, which can generate additional counter movements with different frequencies that cause force peaks during exercising [4]. Due to the limited studies and laboratories that use this device, all the relevant studies that have used this device for multiple purposes are included in this review.

### 4.1. Leg Press Used for Testing and Acute Responses

Five studies used a leg press device as a testing device only [11,12] or in combination for testing and determining the acute effects after a strength loading protocol [11,13,14,15]. In the study of Sedliak et al. [11], leg presses were used to test the bilateral MVC force before and after the training program. In this study, acute responses after bilateral isokinetic leg extensions were monitored. Except for these two studies, a leg press was used for both testing and as an acute loading protocol in the remaining studies. Altogether, when summarizing all these studies, all possible modes were used for testing and acute loading, including isometric, isokinetic, isoinertial (constant), and isokinetic with SSL stimuli. Only two studies used this device to directly compare acute responses after isokinetic strength training with SSL stimuli and without them [14,15]. Kovárová et al. [14] compared the acute responses of the isokinetic bilateral strength protocol with SSL stimuli and the isoinertial protocol (75% 1RM) on bone metabolism outcomes (bone alkaline phosphatase and sclerostin). Their results indicate no significant effect of any of the strength protocols. It should be noted that the results may be hindered by the number of subjects in the study, which was relatively low (*n* = 7), and the selected markers of bone metabolism; moreover, for minor changes, other parameters could be more appropriate (e.g., β-CTX, P1NP, and others) [24]. In another study, Vajda et al. [15] also compared acute responses after isokinetic bilateral strength training, including SSL stimuli and isoinertial (constant) resistance (75% 1RM), in pre- and postmenopausal women. The results indicated possible different acute responses of muscle force, RFD, and hormonal concentrations between pre- and postmenopausal women after the protocol with SSL and isoinertial training. MVC and RFD were significantly decreased after the protocol with SSL in premenopausal women and significantly decreased in postmenopausal women after the isoinertial protocol. The hormone concentration was affected after both protocols only in the premenopausal women. A possible explanation may be age-dependent effects because some data showed that middle-aged women react differently to loading strategies (more resistant to fatigue than younger women) [25]. However, this supposition needs to be further examined due to the limited number of studies that have reported isokinetic strength training (whether acute or long-term) alone and because of the unique nature of the SSL stimuli, compared to the traditional training provided to postmenopausal women and other populations.

### 4.2. Leg Press Used for Training and Its Effect on Various Outcomes

Eight studies used leg press devices for training purposes, and unique SSL stimuli were used directly during the training process [16,17,18,19,20,21,22,23]. Two studies directly compared LP strength training with and without SSL stimuli [16,17], five studies compared LP strength training with SSL stimuli and ES (electrical stimulation) training [18,19,20,21,23], and one study also compared LP strength training with SSL stimuli and standard physiotherapeutic training [24].

For instance, Cvečka et al. [16] compared LP strength training with and without SSL stimuli in a group of young men who trained regularly. The results of their study suggest that the group that trained with the unique SSL stimuli achieved almost double the increments in almost all measured outcomes, except for RFD, maximal concentric force, and CMJ %. However, there was no between-group statistical significance in any of the outcomes measured. Similar results were obtained in the study by Kern et al. [17], who also compared LP strength training with and without SLL stimuli in a group of young men who trained regularly. The results suggested no significant differences between the groups in muscular strength or jump and sprint performance. However, only the group with SSL stimuli significantly improved the RFD and 30 m sprint time results and increased the fast muscle fiber diameter. The above studies indicate that using unique SSL stimuli that can generate force peaks may have a more beneficial effect or produce trends toward greater improvements compared to standard stimuli in young males.

The effects of training between LP strength training with SSL stimuli and ES training were only determined in elderly populations. The results from these studies were somewhat similar, with no significant differences between the groups, as shown in Table 3. However, few studies clearly showed the beneficial effects of one training alternative. For instance, Šarabon et al. [19] compared the effects of LP strength training with SSL stimuli and ES training in seniors on static balance. The results suggest that LP strength training with SSL stimuli led to significant CoP velocity improvement in all measured directions as well as anterior–posterior amplitude improvements compared to the ES group, where only the mediolateral CoP velocity was improved. However, no significant differences between groups were reported. In contrast, Zampieri et al. [21] compared LP strength training with SSL stimuli and ES training and showed that the ES group presented significant improvements in almost all measured outcomes compared to the LP SSL group (only chair raise test and 10 m fast walking test). Similarly, another study by Zampieri et al. [23] compared LP strength training with SSL stimuli and ES straining, and the results suggested that only the ES group presented significant improvements in isometric MVC torque, increased myofiber and mitochondria size, and upregulated IGF1 pan, IGF-1a, IGF-1b, and IGF-1c isoforms. The isokinetic LP SSL group only significantly induced IGF1b isoforms and significantly improved the chair raise test. Only one study [22] was focused on comparing the potential differences between LP strength training with SSL stimuli and standard physiotherapy training. As shown in Table 3, both groups improved all measured outcomes, with no significant differences between the groups.

In the above studies, different training adaptations can be seen after performing LP strength training with unique SSL stimuli. Similar training effects with a positive trend for the LP SSL group were recorded in young males [16] and athletes [17]; however, more variable training effects favoring one or the other approach were achieved in the older population. It should also be noted that only the ES protocol was performed in the senior population; thus, direct comparison of strength training with and without SSL cannot be performed.

Altogether, the studies show that using an LP device with or without SSL stimuli seems to be a very useful alternative because it offers several modes that can be adjusted according to the subject’s needs (i.e., training and testing mode—isokinetic, isometric, isoinertial, SSL mode, bilateral or unilateral adjustment). As shown in Table 3, except for two studies, only an older population was included. This finding suggests that the mentioned LP device with SSL stimuli may be a suitable alternative for the rehabilitation process, which is currently very complex, and strength training overall has its own place in the modern physiotherapy approach [26]. This finding has been documented by numerous research studies, such as the inclusion of strength training after total knee arthroplasty [27,28,29]. One of the included studies in our review (described in detail above) also examined the effect of LP strength training with SSL stimuli and standard physiotherapy training [24] in patients with total knee arthroplasty. Although no significant differences between groups were noted, LP with SSL stimuli appears to be a feasible option in such patients. Linear motor-driven leg press dynamometers with or without SSL stimuli seem to be very useful and safe devices for multipurpose focus for testing, training, and rehabilitation. From the selected studies in this review, it seems that more beneficial effects (trend) of LP strength training with SSL stimuli were observed in young males and athletes and comparable or ambiguous effects were observed in older populations or in rehabilitation patients.

### 4.3. Limitations

Several limitations need to be mentioned. First, only a scoping review without a meta-analysis was performed due to the lack of studies and variable outcomes that were measured. There are only two prototypes of this device; thus, finding studies from a wide range of authors across the world is nearly impossible, which prevents the performance of a meta-analysis with the same or at least comparable outcomes. Additional short- and long-term studies focused on SSL stimuli during exercising in young and older populations as well as rehabilitation patients are required to draw more specific conclusions.

## 5. Conclusions and Practical Applications

This review article is focused on a linear motor-driven leg press dynamometer with a multipurpose focus and unique serial stretch loading stimuli that can generate force peaks at different frequencies. This review shows the usability of the device for testing to assess the acute and long-term effects after strength training using SSL stimuli. The tables, results, and discussion sections show that this device is useful and has a multipurpose focus because it can also be used in rehabilitative patients. Logical comparisons of the selected studies indicate the potential advantage (at trend level) of LP with SSL stimuli in young male and athletes where it was demonstrated that those who trained with SSL stimuli achieved greater adaptation effect in muscular force, jumping, and sprinting capabilities compared to traditional resistance. However, the positive but ambiguous effects relative to other forms of training were noted in older populations, which indicate that various methods or approaches may improve/restore physical functions in the elderly to a similar extent. Overall, using this device and all its modes, especially SSL stimuli, seems to be a very interesting alternative that can be added to the training process in order to improve physical performance in the young or athlete population and rehabilitation process.

## Figures and Tables

**Table 1:** Qualitative evaluation of the selected studies. Training and acute/testing studies are presented.

Type of Studies	Acute/Testing Studies					Training Studies							
Quality Criteria (PEDro)	Sedliak et al. [11]	Billy et al. [12]	Sedliak et al. [13]	Kovárová et al. [14]	Vajda et al. [15]	Cvečka et al. [16]	Kern et al. [17]	Kern et al. [18]	Šarabon et al. [19]	Cvečka et al. [20]	Zampieri et al. [21]	Billy et al. [22]	Zampieri et al. [23]
Were subject randomly allocated to groups?	1	0	1	0	1	1	1	0	1	0	0	1	1
Was allocation concealed?	0	0	0	0	0	0	0	0	0	0	0	1	0
Were the groups similar at baseline?	1	0	1	0	0	0	1	0	1	1	1	1	1
Were measures of at least one key outcome obtained from more than 85% of the subjects?	1	1	1	1	1	1	1	1	1	1	1	1	1
Did all subjects receive the treatment or control condition as allocated (or at least one key outcome was analyzed by “intention to treat”)	1	0	1	1	1	1	1	1	1	1	1	1	1
Was there between-group statistical comparison for at least one key outcome?	1	0	1	1	1	1	1	0	1	1	1	1	1
Did the study provides both point measures and measures of variability for at least one key outcome?	1	1	1	1	1	1	1	1	1	1	1	1	1
Score out of 7	6	2	6	4	5	5	6	3	6	5	5	7	6

Note: Only relevant questions without additions are shown in the table, training studies = long-term application of leg press strength training with SSL stimuli, acute/testing studies = leg press device used for testing purposes or to assess acute responses after strength loading with SSL stimuli.

**Table 2:** Acute/testing studies using a leg press dynamometer.

Study	Sample	Design	Measures	Intervention	Results
Sedliak et al. [11]	Untrained young males (*n* = 25) Morning group (*n* = 11, 23 ± 2 years) Afternoon group (*n* = 7, 24 ± 4 years) Control group (*n* = 7, 24 ± 3 years)	Randomized controlled trial Leg press dynamometer used for testing and acute loading protocol	Isometric bilateral MVC peak force on a leg press device Muscle biopsies - mean QF CSA - mean fiber CSA - cortisol - testosterone Cell signaling - p70S6Thr421/Ser424 - rpS6 - p38 MAPK - p70S6Thr389 - eEF2 - Erk1/2	11-week training Acute loading protocol - 5 sets of 10 reps of isokinetic bilateral leg extension - speed of the pedals was 0.2 m/s Training protocol - 2 x/week - main muscle groups: leg presses, knee extensions and flexions and 5 upper body and core exercises - 3 to 4 sets with 8 to 12 reps - intensity from 40 to 80% of 1RM	Isometric bilateral MVC force sig. increased in the morning (16.9%, *p* < 0.01) and afternoon groups (15.2%, *p* < 0.05) Mean QF sig. increased in the morning (8.8%, *p* < 0.001) and afternoon groups (11.9%, *p* < 0.05) Fiber CSA sig. increased in the morning (21%, *p* < 0.05) and afternoon groups (18%, *p* < 0.05) Cortisol level sig. decreased in all three groups (*p* < 0.05) p70S6Thr421/Ser424 sig. increased in the morning group after training (*p* < 0.01) rpS6 and p38 MAPK sig. increased after acute loading in both groups before and after training (*p* < 0.05) No sig. changes in thep70S6Thr389, eEF2, and Erk1/2
Billy et al. [12]	Elderly patients with knee osteoarthritis (*n* = 75, M: 24, F: 51, 67.5 ± 7.3 years)	Cross sectional exploratory study	Leg press device isometric testing - MVC force Isokinetic testing - concentric and eccentric - velocity of 0.1 m/s Unilateral testing Knee extension MVC force TUG Stair test	No intervention	All strength measures provide satisfactory predictive models for the stair test (21.5%, *p* = 0.009) but not TUG (11.9%, *p* = 0.068) TUG (r = −0.29–−0.48, *p* = 0.01–0.001) and stair test (r = −0.40–−0.63, *p* = 0.001) were sig. correlated with all strength measures (moderate effects)
Sedliak et al. [13]	Young active males (*n* = 22) - Morning group: (*n* = 11, 25.0 ± 4.0 years) - Afternoon group: (*n* = 11, 24.0 ± 3.0 years)	Randomized controlled trial Two groups pre/post design - Acute effects	Isometric bilateral leg extension with MVC peak force on a leg press device and - 3 to 4 s ec of contraction duration Muscle biopsies	Isokinetic bilateral leg extension with the MVC protocol on a leg press device -pedal speed of 0.2 m/s 5 sets and 10 reps	Muscle force sig. declined in the morning (−39%, *p* < 0.001) and afternoon groups (−38%, *p* < 0.001) after the strength protocol P70S6K Thr389 (*p* < 0.05) and Thr421/Ser424 and rpS6 sig. increased (*p* < 0.001) after strength protocol in both groups The afternoon group showed a sig. decrease post-loading (*p* < 0.05) and sig. increase (*p* < 0.01) in eEF2 and p38 MAPK, respectively
Kovárová et al. [14]	Young women (*n* = 7, 22.7 ± 1.9 years)	Design not specified (based on the methodology RCT) Acute effects	Bone metabolism - bone alkaline phosphatase - Sclerostin	Isokinetic bilateral LP strength protocol with SSL - velocity 50 and 40 cm·s^−1^ - every 20 mm short interruption incorporated - frequency of force peaks was 10 Hz - 6 sets and 6 reps with 2-min rest time Isoinertial (constant resistance) bilateral LP strength protocol - 75% of 1RM - 6 sets with 6 reps and 2 min rest time CON protocol without application of specific stimuli	No sig. effect of isoinertial or isokinetic strength protocol with SSL on bone alkaline phosphatase and sclerostin
Vajda et al. [15]	Sedentary women 2 groups - premenopausal women (*n* = 8, 22.6 ± 2.5 years) - postmenopausal women (*n* = 9, 55.2 ± 5.7 years)	Randomized controlled trials Acute effects	Isometric bilateral MVC peak force and RFD on a leg press device Isokinetic bilateral power output -velocity of 20 cm·s^−1^ Testosterone, estradiol, cortisol, growth hormone, lactate	Isokinetic bilateral LP resistance loading including SSL - velocity 50 cm·s^−1^ with short interruptions lasting 10 ms every 20 mm - frequency 10 Hz - 6 sets and 6 reps with 90 s rest time Isoinertial (constant) bilateral resistance loading without SSL - 75% of 1RM - 6 sets and 6 reps with 90 s rest time	MVC (from 2587.63 ± 746.77 N to 2035.01 ± 666.27 N, *p* < 0.05) and RFD (from 6.91 ± 1.34 Nm to 5.38 ± 1.28 Nm, *p* < 0.05) sig. decreased after LP with SSL protocol in premenopausal women MVC (from 1722.02 ± 520.56 N to 1561.61 ± 435.48 N, *p* < 0.05) and RFD (from 4.81 ± 1.17 Nm to 3.92 ± 0.99 Nm, *p* < 0.05) sig. decreased after LP with isoinertial resistance in postmenopausal women Testosterone, estradiol, and cortisol sig. decreased after both protocols in premenopausal women (all *p* < 0.05) Testosterone and estradiol after LP with isoinertial resistance, and cortisol after LP with SSL were sig. larger (all *p* < 0.05) in premenopausal compared to postmenopausal women

Note: *n* = sample size, MVC = maximal voluntary contraction, s = seconds, m/s = meters per seconds, Hz = hertz, sig. = significant, RCT = randomized controlled trial, LP = leg press, SSL = serial stretch loading, 1RM = one repetition maximum, CSA = cross sectional area, RFD = rate of force development, N = Newton, Nm = Newton meter, M = male, F = female, TUG = timed up and go test.

**Table 3:** Long-term training studies using a leg press dynamometer during strength training.

Study	Sample	Design	Measures	Intervention	Results
Cvečka et al. [16]	Young well-trained males Isokinetic LP SSL group (*n* = 17, 23.3 ± 2.6 years) Isokinetic LP group (*n* = 16, 22.6 ± 2.5 years)	Randomized controlled trial Two groups pre/post design	Isometric bilateral MVC force on a leg press device Isokinetic bilateral maximal and mean force in concentric and eccentric phase of leg press exercise Isometric bilateral RFD (200 ms) on a leg press device CMJ height	Duration 8 weeks Trained 3 x/week Isokinetic bilateral LP SSL group - 6 sets and 6 reps - 0.3 m/s and 0.2 m/s extension and flexion velocity, respectively - 5 mm SSL counter movements Isokinetic bilateral LP group - 9 sets and 6 reps (higher volume compensate for time loss due to SSL mode duration in the LP SSL group) - 0.3 m/s and 0.2 m/s extension and flexion velocity, respectively	Both groups showed sig. increases in MVC (LP SSL: 48.1%, *p* < 0.01; LP group: 24.8%, *p* < 0.01) RFD (LP SSL: 37.9%, *p* < 0.05; LP group: 31.4%, *p* < 0.05) and maximal concentric force (LP SSL: 45.4%, *p* < 0.01; LP group: 47.0%, *p* < 0.01). Mean concentric force sig. increased only in LP SSL (47.5%, *p* < 0.01) Maximal eccentric force sig. increased in both groups (LP SSL: 43.6%, *p* < 0.01; LP group: 24.7%, *p* < 0.01) Mean eccentric force sig. increased in both groups (LP SSL: 43.5%, *p* < 0.01; LP group: 24.9%, *p* < 0.05) CMJ sig. increased only in the LP SSL group (7.2%, *p* < 0.05) Isokinetic LP SSL achieved almost double the % increments in MVC, mean concentric force, maximal eccentric force and mean eccentric force compared to the isokinetic LP group only RFD, maximal concentric force and CMJ % improvements were similar between the groups
Kern et al. [17]	Young male athletes (*n* = 29, 22.95 ±.2 years) Isokinetic LP SSL group (23.1 ± 2.7 years) Isokinetic LP group (22.6 ± 3.9 years)	Randomized controlled trial Two groups pre/post design	Isometric unilateral MVC force and RFD (0–50 ms) on a leg press device SJ height 30-m sprint time Muscle biopsies - fiber type distribution and diameter Gene expression	Duration 8 weeks Trained 3 x/week Unilateral or bilateral training is not defined Concentric velocity was 0.3 m/s and eccentric one 0.2 m/s Isokinetic LP SSL group - 6 sets of 6 reps with maximal effort including short countermovement (0.5 cm) every 2 cm Isokinetic LP group - standard isokinetic mode with 6 sets and 8 reps (compensate time difference compared to the other group) 2 min rest time between the sets	Both groups showed significantly improved isometric unilateral MVC force (LP SSL: 48.1%, *p* < 0.01; LP group: 24.8%, *p* < 0.01) Only the LP SSL group showed sig. improvements in the RFD (30.2%, *p* < 0.001), SJ height (7.4%, *p* < 0.005) as well as 30-m sprint time (−1.3%, *p* < 0.05) No significant differences between the groups in the strength outcomes, jump and sprit time Only the LP SSL group significantly increased fast muscle fiber diameter (9%, *p* < 0.001) - No changes in the LP group only - Changes were significantly higher in the LP SSL group compared to the LP group only (*p* < 0.001) LP SSL group showed sig. increases in IGF-1Ec (2-fold change, *p* < 0.05) and PGC-1α (228%, *p* < 0.05) Significant downregulation of myostatin occurred only in the LP SSL group (4-fold change, *p* < 0.0005)
Kern et al. [18]	Seniors (gender not defined)Group 1 (Vienna): 2 subgroups - Isokinetic LP SSL group (*n* = 16, 74.93 ± 5.48 years) - ES group (*n* = 16, 73.20 ± 6.56 years) Group 2 (Bratislava): 2 subgroups -Isokinetic LP SSL group (*n* = 9, 71.12 ± 3.34 years) - ES group (*n* = 9, 70.41 ± 3.74 years)	Randomized controlled trial Four groups pre/post design	Unilateral knee extension - Isometric MVC force and RFD on a force chair 10 m fasted walking Chair raise TUG Stair test Dynamic balance Muscle biopsies - myofibers diameter	8–10 weeks of training (10 in Group 1, 8 in Group 2) Bilateral training Two subgroups (isokinetic LP SSL groups) performed a ST on the LP device with SSL mode One subgroup from each group (ES groups) performed home-based electrical stimulation Detailed training program is not specified	Group 1: LP SSL subgroup showed sig. improvements in all functional tests except for MVC force. ES subgroup showed sig. improvements in all functional tests except for dynamic balance Group 2: LP SSL subgroup showed sig. improvement in only the chair raise test (from 12.52 ± 1.98 to 10.12 ± 1.41 s, *p* = 0.041) while others remained unchanged. ES subgroup showed sig. improvements in also chair rise test (from 13.12 ± 2.60 to 11.25 ± 1.66 s, *p* = 0.018) Both groups and their subgroups showed sig. increases in myofiber diameter
Šarabon et al. [19]	Sedentary seniors (gender not defined) 74.3 ± 7.0 years Three groups: - Isokinetic LP SSL group (*n* = 28) - ES group (*n* = 27) - CON group (*n* = 19)	Randomized controlled trial Three groups pre/post design	30 s static balance - average velocity, amplitude, and frequency of CoP - total, medial-lateral, anterior-posterior direction	Duration 9 weeks Trained 3 x/week Bilateral training Isokinetic LP SSL group - velocity of the pedals was 0.3 m/s and 0.2 m/s for concentric and eccentric phase, respectively - every 8 mm was interrupted by a short stop that resulted in force peaks - 2–3 sessions/week - 4–5 sets/session - time/set from 8 to 14 s - ES group: anterior thigh stimulation (both legs) with frequency of 60 Hz - 45 contractions (3 × 15 reps, 2 sessions in the first 2 weeks) - 75 contractions (from week 3 to 9) CON group: continued in their normal daily activities	The Isokinetic LP SSL group showed sig. improvements in CoP velocity in anterior-posterior (from 14.4 ± 1.5 to 11.4 ± 1.1 mm/s, *p* < 0.05), medial-lateral (from 7.5 ± 0.7 to 6.1 ± 0.5 mm/s, *p* < 0.05) and total direction (from 17.6 ± 1.6 to 15.2 ± 1.2 mm/s, *p* < 0.05) as well as anterior-posterior amplitude (from 5.6 ± 0.5 to 4.9 ± 0.5 mm, *p* < 0.05) The ES group showed sig. improvements in medial-lateral CoP velocity (from 6.9 ± 0.7 to 5.6 ± 0.4 mm/s, *p* < 0.05) The CON group sig. worsened CoP anterior-posterior velocity (from 14.6 ± 1.7 to 16.1 ± 1.5 mm/s, *p* < 0.05)
Cvečka et al. [20]	Sedentary seniors Gender and age are not defined Two groups: - Isokinetic LP SSL group - ES group	Randomized controlled trial Two groups pre/post design	Isometric MVC torque on a chair dynamometer - bilateral or unilateral testing is not defined Chair rising test TUG 10 m walk test	Duration 8 weeks Bilateral or unilateral training is not defined Isokinetic LP SSL group - frequency of 16 and 14 Hz - 5 sets with 12–14 s of contraction time - 3 x/week ES group - knee extensors ES - 3 x/week - 3 sets of 10 min (first 2 weeks 3 sets of 6 min)	The LP SSL group showed sig. improvements in MVC torque (from 222 to 236 Nm, *p* < 0.05), chair rising test (from 12.5 to 10.4 s, *p* < 0.05), TUG (from 6.29 to 5.68 s, *p* < 0.05), 10 m walk test (from 5.06 to 4.80 s, *p* < 0.05), and postural stability test (data not shown) The ES group showed sig. improvements in MVC torque (from 232 to 248 Nm, *p* < 0.05), chair rising test (from 13.10 to 10.80 s, *p* < 0.05), TUG (from 7.61 to 6.96 s, *p* < 0.05), and 10 m walk test (from 5.96 to 5.52 s, *p* < 0.05) No sig. differences between the groups
Zampieri et al. [21]	Sedentary seniors (M/F) Isokinetic LP SSL group (*n* = 9, M = 5, F = 4, 71.8 ± 7.1 years) ES group (*n* = 16, M = 8, F = 8, 70.6 ± 2.8 years)	Randomized controlled trial Two groups pre/post design	Isometric MVC torque on a chair dynamometer Functional tests using “SFT battery” - TUG - Chair raise - 10 m habitual walking test - 10 m fast walking test Muscle biopsy including myofiber diameter Unilateral or bilateral testing is not specified	Duration 9 weeks Isokinetic LP SSL group - 3 x/week ES group - 3 x/week Detailed training program is not specified in both groups Unilateral or bilateral training is not specified	The isokinetic LP SSL group showed sig. improvements in chair rise test (from 10.95 ± 1.75 to 9.54 ± 1.92 s, *p* < 0.05) and 10 m fast walking test (from 1.90 ± 0.19 to 2.01 ± 0.23 s, *p* < 0.005) The ES group showed sig. improvements in isometric MVC torque (from 1.42 ± 0.34 to 1.51 ± 0.38 Nm, *p* < 0.05), TUG (from 8.42 ± 1.95 to 7.04 ± 1.09 s, *p* < 0.0005), chair rise test (from 13.85 ± 3.33 to 10.53 ± 3.63 s, *p* < 0.005), 10 m habitual walking test (from 1.20 ± 0.19 to 1.26 ± 0.18 s, *p* < 0.05) and 10 m fast walking test (from 1.58 ± 0.28 to 1.66 ± 0.24 s, *p* < 0.05) The isokinetic LP SSL group showed sig. decreases in slow (from 55.43 ± 17.33 to 53.12 ± 16.06 μm, *p* < 0.001) and fast type myofiber diameter (from 48.96 ± 16.18 to 46.43 ± 15.96 μm, *p* < 0.001) The ES group showed sig. decreases in slow type myofiber diameter (from 50.30 ± 14.78 to 48.48 ± 16.67 μm, *p* < 0.001) but sig. increases in the fast type myofiber diameter (from 46.53 ± 14.04 to 47.54 ± 15.79 μm, *p* < 0.001)
Billy et al. [22]	Sedentary seniors (M/F) Total knee arthroplasty Isokinetic LP SSL group (*n* = 26, M = 9, F = 17, 64.9 ± 6.0 years) Physiotherapy group (*n* = 29, M = 9, F = 20, 68.3 ± 6.7 years)	Randomized controlled trial Two groups pre/post design	Isometric unilateral MVC peak force of leg extension on a leg press device Isometric unilateral MVC torque of knee extension on a force chair TUG Stair test Pain and function Active and passive range of motion	Duration 6 weeks Trained 2 x/week Unilateral training-involved and uninvolved leg Isokinetic LP SSL group - 4 to 6 sets of 22 to 25 s with SSL during concentric phase interrupted by a countermovement (1 to 2 cm backward) Physiotherapy group - physiotherapy training included cycling, manual and soft tissue therapy, ROM-exercises, isometric and dynamic strengthening exercises, and gait-retraining exercises - 1 to 3 sets of 10 to 15 reps with individualized intensity - duration of 1 session was 30 min	The LP SSL group showed sig. improvements in MVC force on a leg press device with involved leg (from 8.9 ± 0.77 to 10.3 ± 1.06 N/kg, *p* < 0.05), MVC on force chair with involved (from 0.8 ± 0.06 to 1.0 ± 0.09 Nm/kg, *p* < 0.01) and uninvolved leg (from 1.2 ± 0.09 to 1.2 ± 0.11 Nm/kg, *p* < 0.01) - The LP SSL group showed sig. improvements in all other functional outcomes Physiotherapy group showed sig. improvements in MVC force on a leg press device with involved leg (from 6.7 ± 0.54 to 9.1 ± 0.70 N/kg, *p* < 0.05), MVC on force chair with involved (from 0.7 ± 0.06 to 0.9 ± 0.06 Nm/kg, *p* < 0.00) and uninvolved leg (from 1.1 ± 0.08 to 1.2 ± 0.07 Nm/kg, *p* < 0.01) - The PT group showed sig. improvements in all other functional outcomes No sig. differences between the groups after training were recorded in any of the examined outcomes
Zampieri et al. [23]	Sedentary seniors (M/F) Isokinetic LP SSL group (*n* = 7, M = 4, F = 3, 70.1 ± 2.9 years) ES group (*n* = 10, M = 5, F = 5, 71.4 ± 7.1 years)	Randomized controlled trial Two groups pre/post design	Isometric MVC torque on a force chair Time to raise from a chair Muscle biopsies Gene expression Mitochondrial dynamics Unilateral or bilateral testing is not specified	Duration 9 weeks Trained 2–3 x/week Isokinetic LP SSL group - intensity approximately 90% of MVC - detailed training program of leg press training is not defined ES group - ES of the thigh quadriceps musculature of both legs at 60 Hz by 3.5-s train of impulses with 4.5-s off intervals - intensity: approximately 40% of MVC Unilateral or bilateral training is not specified	The isokinetic LP SSL group showed sig. improvements in chair raise test (*p* = 0.050) but no sig. changes in MVC torque The ES group showed sig. improvements in MVC torque (*p* = 0.026) and chair raise test (*p* = 0.036) The ES group showed sig. increases in myofiber size (from 49.16 ± 15.80 to 51.01 ± 16.38 μm, *p* < 0.0001) The isokinetic LP SSL group showed sig. decreases in myofiber size (from 57.87 ± 19.17 to 55.21 ± 18.13 μm, *p* < 0.0001) Only the ES group showed sig. decreases in the atrophy factor (*p* = 0.031) The ES group showed sig. upregulation of IGF1 pan (*p* = 0.001), IGF-1a (*p* = 0.001), IGF-1b (*p* = 0.014), IGF-1c isoforms (*p* = 0.013) The Isokinetic LP SSL group showed sig. induction of IGF1b isoforms (*p* = 0.002) Only the ES group showed sig. increases in mitochondria size (from 72.3 ± 1.9 to 80.4 ± 2.5 μm^2^, *p* = 0.009), although the mitochondria number sig. decreased (from 48.3 ± 1.3 to 38.6 ± 1.2 μm^2^, *p* = 0.0001) - No changes in the isokinetic LP SSL group

Note: *n* = sample size, MVC = maximal voluntary contraction, CMJ = countermovement jump height, SJ = squat jump height, ES = electrical stimulation group, CoP = center of pressure, μm = micrometer, s = seconds, m/s = meters per seconds, sig. = significant, RFD = rate of force development, N = Newton, Nm = Newton meter, M = male, F = female, TUG = timed up and go test.

## References

[1] Yong-Seok J. (2015). Usefulness of measuring isokinetic torque and balance ability for exercise rehabilitation. J. Exerc. Rehabil..

[2] Mavroidis C., Nikitczuk J., Weinberg B., Danaher G., Jensen K., Pelletier P., Prugnarola J., Stuart R., Arango R., Leahey M. (2005). Smart portable rehabilitation devices. J. Neuroeng. Rehabil..

[3] Gassert R., Dietz V. (2018). Rehabilitation robots for the treatment of sensorimotor deficits: A neurophysiological perspective. J. Neuroeng. Rehabil..

[4] Hamar D. (2015). Universal linear motor driven Leg Press Dynamometer and concept of Serial Stretch Loading. Eur. J. Transl. Myol.-Basic Appl. Myol..

[5] Wang E., Nyberg S.K., Hoff J., Zhao J., Leivseth G., Tørhaug T., Husby O.S., Helgerud J., Richardson R.S. (2017). Impact of maximal strength training on work efficiency and muscle fiber type in the elderly: Implications for physical function and fall prevention. Exp. Gerontol..

[6] Caserotti P., Aagaard P., Larsen J.B., Puggaard L. (2008). Explosive heavy-resistance training in old and very old adults: Changes in rapid muscle force, strength and power. Scand. J. Med. Sci. Sports.

[7] Alcazar J., Csapo R., Ara I., Alegre L.M. (2019). On the Shape of the Force-Velocity Relationship in Skeletal Muscles: The Linear, the Hyperbolic, and the Double-Hyperbolic. Front. Physiol..

[8] Tricco A.C., Lillie E., Zarin W., O’Brien K.K., Colquhoun H., Levac D., Moher D., Peters M.D.J., Horsley T., Weeks L. (2018). PRISMA Extension for Scoping Reviews (PRISMA-ScR): Checklist and Explanation. Ann. Intern. Med..

[9] Maher G.C., Sherrington C., Herbert D.R., Moseley M.A., Elkins M. (2003). Reliability of the PEDro scale for rating quality of randomized controlled trials. Phys. Ther..

[10] Kümmel J., Kramer A., Giboin L.-S., Gruber M. (2016). Specificity of Balance Training in Healthy Individuals: A Systematic Review and Meta-Analysis. Sports Med..

[11] Sedliak M., Zeman M., Buzgó G., Cvecka J., Hamar D., Laczo E., Okuliarova M., Vanderka M., Kampmiller T., Häkkinen K. (2018). Morphological, molecular and hormonal adaptations to early morning versus after-noon resistance training. Chronobiol. Int..

[12] Billy W., Sarabon N., Löfler S., Franz C., Wakolbinger R., Kern H. (2019). Relationship between strength parameters and functional performance testsin patients with severe knee osteoarthritis. PM R..

[13] Sedliak M., Zeman M., Buzgó G., Cvečka J., Hamar D., Laczo E., Zelko A., Okuliarová M., Raastad T., Nilsen T.S. (2013). Effect of time of day on esistance exercise-induced anabolic signaling in skeletal muscle. Biol. Rhythm Res..

[14] Kovárová J., Hamar D., Sedliak M., Cvečka J., Schickhofer P., Böhmerová Ľ. (2015). Acute Response of Bone Metabolism to Various Resistance Exercises in Women. AFEPUC.

[15] Vajda M., Kovarova J., Okuliarova M., Cvecka J., Schickhofer P., Bohmerova L. (2017). Acute hormonal and neuromuscular response to various loading in young pre- and middle-aged postmenopausal women. Gazz. Med. Ital. Arch. Sci. Med..

[16] Cvecka J., Hamar D., Trimmel L., Vogelauer M., Bily W. (2009). Einfluss von serial stretch loading auf die Effektivität des isokinetischen. BAM.

[17] Kern H., Pelosi L., Coletto L., Musaro A., Sandri M., Vogelauer M., Trimmel L., Cvecka J., Hamar D., Kovarik J. (2011). Atrophy/hypertrophy cell signaling in muscles of young athletes trained with vibration-al-proprioceptive stimulation. Neurol. Res..

[18] Kern H., Loefler S., Hofer C., Vogelauer M., Burggraf S., Grim-Stieger M., Cvecka J., Hamar D., Sarabon N., Protasi F. (2012). FES Training in Aging: Interim results show statistically significant improvements in mobility and muscle fiber size. Eur. J. Transl. Myol..

[19] Nejc S., Loefler S., Cvecka J., Sedliak M., Kern H. (2013). Strength training in elderly people improves static balance: A randomized controlled trial. Eur. J. Transl. Myol..

[20] Cvecka J., Tirpakova V., Sedliak M., Kern H., Mayr W., Hamar D. (2015). Physical activity in elderly. Eur. J. Transl. Myol..

[21] Zampieri S., Mosole S., Löfler S., Fruhmann H., Burggraf S., Cvečka J., Hamar D., Sedliak M., Tirptakova V., Šarabon N. (2015). Physical exercise in Aging: Nine weeks of leg press or electrical stimulation training in 70 years old sedentary elderly people. Eur. J. Transl. Myol..

[22] Bily W., Franz C., Trimmel L., Loefler S., Cvecka J., Zampieri S., Kasche W., Sarabon N., Zenz P., Kern H. (2016). Effects of Leg-Press Training with Moderate Vibration on Muscle Strength, Pain, and Function After Total Knee Arthroplasty: A Randomized Controlled Trial. Arch. Phys. Med. Rehabil..

[23] Zampieri S., Mammucari C., Romanello V., Bardberi L., Pietrangelo L., Fusella A., Mosole S., Gherardi G., Höfer C., Löfler S. (2016). Physical exercise in aging human skeletal muscle increases mitochondrial calcium uniporter expression levels and affects mitochondria dynamics. Physiol. Rep..

[24] Scott J.P., Sale C., Greeves J.P., Casey A., Dutton J., Fraser W.D. (2011). The role of exercise intensity in the bone metabolic response to an acute bout of weight-bearing exercise. J. Appl. Physiol..

[25] Avin G.K., Law F.L. (2011). Age-related differences in muscle fatigue vary by contraction type: A meta-analysis. Phys. Ther..

[26] Shaw I., Shaw S.B., Brown A.G., Shariat A. (2016). Review of the Role of Resistance Training and Muscu- loskeletal Injury Pre-vention and Rehabilitation. Gavin J. Orthop. Res. Ther..

[27] Nguyen Ch Lefèvre-Colau M.M., Poiraudeau S., Rannou F. (2016). Rehabilitation (exercise and strength training) and osteoar-thritis: A critical narrative review. Ann. Phys. Rehabil. Med..

[28] Jakobsen T.L., Kehlet H., Husted H., Petersen J., Bandholm T. (2014). Early Progressive Strength Training to Enhance Recovery After Fast-Track Total Knee Arthroplasty: A Randomized Controlled Trial. Arthritis Care Res..

[29] Husby S.V., Foss A.O., Husby S.O., Winther B.S. (2018). Randomized controlled trial of maximal strength training vs. standard rehabilitation following total knee arthroplasty. Eur. J. Phys. Rehabil. Med..

